# Nanocarriers for Medical Ozone Delivery: A New Therapeutic Strategy

**DOI:** 10.3390/nano15151188

**Published:** 2025-08-03

**Authors:** Manuela Malatesta, Flavia Carton

**Affiliations:** 1 Department of Neurosciences, Biomedicine and Movement Sciences, University of Verona, 37134 Verona, Italy; 2 Center for Medical Sciences (CISMed), University of Trento, 38122 Trento, Italy; flavia.carton@unitn.it; 3 Department of Cellular, Computational and Integrative Biology (CIBIO), University of Trento, 38123 Trento, Italy

**Keywords:** ozone nanodelivery, oxidative therapy, antiseptic treatment, anticancer treatment, pro-regenerative treatment, liposomes, nanobubbles, nanohydrogels, niosomes, polymeric nanoparticles

## Abstract

Ozone (O_3_) occurs in nature as a chemical compound made of three oxygen atoms. It is an unstable, highly oxidative gas that rapidly decomposes into oxygen. The therapeutic use of O_3_ dates back to the beginning of the 20th century and is currently based on the application of low doses, inducing a moderate oxidative stress that stimulates the antioxidant cellular defenses without causing cell damage. Low O_3_ doses also induce anti-inflammatory and regenerative effects, and their anticancer potential is under investigation. In addition, the oxidative properties of O_3_ make it an excellent antibacterial, antimycotic, and antiviral agent. Thanks to these properties, O_3_ is currently widely used in several medical fields. However, its chemical instability represents an application limit, and ozonated oil is the only stabilized form of medical O_3_. In recent years, novel O_3_ formulations have been proposed for their sustained and more efficient administration, based on nanotechnology. This review offers an overview of the nanocarriers designed for the delivery of medical O_3_, and of their therapeutic applications. The reviewed articles demonstrate that research is active and productive, though it is a rather new entry in the nanotechnological field. Liposomes, nanobubbles, nanoconstructed hydrogels, polymeric nanoparticles, and niosomes were designed to deliver O_3_ and have been proven to exert antiseptic, anticancer, and pro-regenerative effects when administered in vitro and in vivo. Improving the therapeutic administration of O_3_ through nanocarriers is a just-started challenge, and multiple prospects may be foreseen.

## 1. Introduction

Ozone (O_3_) is a chemical compound made of three oxygen atoms and naturally occurring in the atmosphere; it is highly unstable and rapidly decomposes into oxygen (O_2_), having a half-life of about 40 min at 20 °C [1].

O_3_ was described as an individual chemical substance in 1839 by Christian Friedrich Schönbein, who gave it the name from the Greek ὄζειν (ozein, meaning to smell) [2]. In fact, O_3_ was first detected in 1785 by Martinus van Marum, a Dutch physician who noticed O_3_ presence from the typical smell acquired by air that had been passed by an electric spark [3]. The first demonstration of the oxidizing power of O_3_ was reported by Schönbein, who showed that O_3_ is able to react with both inorganic [4] and organic compounds [5], thus being potentially harmful to human and animal health [6]. In the 1870s, Fox [7] and Kellogg [8] described the disinfectant action of O_3_ against microorganisms, but the use of O_3_ in medicine was made possible only in the 1900s, when O_3_ generators were produced and sold by the Tesla Ozone Company (Nikola Tesla’s US Patent no. 568,177, 22 September 1896). It was then practically established that O_3_ may effectively be applied for curing a wide spectrum of pathological conditions, such as infected wounds and chronic inflammatory diseases (reviews in [9,10,11]). The advent of O_3_-resistant plastics (in the 1950s) and the commercial availability of O_3_ generators that precisely measure gas concentrations in real time (in the 1990s) were crucial for O_3_ to become increasingly accepted and growingly applied as an auxiliary medical treatment [12,13,14].

In medicine, O_3_ is usually administered to patients in the form of a gaseous O_2_-O_3_ mixture or as ozonated water or oil. Treatment may be systemic by autohemotherapy (i.e., ozonating ex vivo a small aliquot of blood and reinfusing it into the bloodstream) or by rectal insufflation, or local by topical application or injection. Gaseous medical O_3_ is prepared using an O_3_ generator that, passing pure O_2_ through a high-voltage electrical discharge, produces the O_2_-O_3_ mixture at a photometrically controlled O_3_ concentration. It is worth noting that medical grade O_2_ must be used for preparing the O_2_-O_3_ mixtures, in order to avoid the formation of potentially toxic nitrated by-products [15]. Ozonated water is produced by bubbling or injecting the O_2_-O_3_ gas mixture into water for a few minutes. Similarly, medical ozonated oil is prepared by continuously bubbling the O_2_-O_3_ mixture into vegetable oils (see below) for days or weeks.

The O_3_ concentration is critical for positive effects to be obtained, and nowadays the O_3_ therapy is based on the low-dose concept, as proposed by Viebahn-Hänsler et al. [16,17]. It was indeed demonstrated that only low O_3_ concentrations (from 10 to 50 µg O_3_/mL O_2_) are therapeutically effective, as they are able to induce a moderate oxidative stress [18] (defined as eustress [19,20]) that stimulates an antioxidant and anti-inflammatory reaction without causing cell damage [21,22].

The knowledge of the molecular pathways involved in the action of O_3_ on biological systems is still incomplete, but growing experimental evidence, in the recent literature, has demonstrated that some of the beneficial effects of O_3_ administration can be mechanistically explained (recent review in [23]). The oxidative stress is counteracted by O_3_ through the activation of Nrf2, which upregulates several antioxidant factors, thus promoting cell survival [24,25] and inhibiting cell death [26,27,28]. The anti-inflammatory effect of O_3_ depends on the modulation of cytokines [29,30,31,32,33,34], the inhibition of matrix metalloproteinases [35], and the increased macrophage phagocytosis [36,37]. These anti-inflammatory effects and the inhibition of cell death are also responsible for the painkilling action of O_3_ [38,39,40,41,42]. The regenerative potential of O_3_ in wound [43,44], bone [45,46], and nerve healing [47,48,49] relies on its anti-apoptotic, pro-proliferative, antioxidant, and anti-inflammatory properties as well as on fibroblast stimulation [50,51,52]. The anticancer action of O_3_ is based on its oxidant and anti-inflammatory properties as well as on its capability to promote apoptosis and inhibit proliferation and migration of tumor cells [37,53,54,55,56].

One of the main limits for the therapeutic efficacy of O_3_ is its intrinsic instability, the half-life of aqueous O_3_ at 35 °C being less than 8 min [57]. Both O_2_-O_3_ gas mixtures and ozonated water are rapidly degraded, require careful handling, and are unsuitable for transport, storage, and extra-hospital treatment. On the contrary, ozonated oil may maintain its properties for up to two years, if appropriately stored [58]. In fact, as first reported by Fenaroli in the early 1900s [59], gaseous O_3_ directly reacts with the double carbon-to-carbon bonds of unsaturated fatty acids, giving rise to stable ozonated derivatives. Ozonated oils for therapeutic purposes are prepared using unsaturated vegetable oils (mainly from olive, sunflower, and sesame), where O_3_ forms ozonides such as 1,2,4-trioxolanes and various peroxides (e.g., hydroperoxides, H_2_O_2_) [60,61,62]. The ozonides represent the active form of O_3_ responsible for the antibacterial, antimycotic, anti-inflammatory, and regenerative effects of the ozonated oils, which are currently used for topical treatments in ophthalmology, dermatology, and dentistry (e.g., reviews in [15,63,64,65,66,67,68]).

## 2. Search Strategy for the Literature

In recent years, novel strategies have been explored using nanotechnologies to improve the administration of O_3_ and prolong its stability as an active molecule in tissues. In the following chapters, we originally review the research on nanostructured formulations designed for efficient and sustained O_3_ delivery, and discuss the efficacy of their biomedical applications.

The scientific literature on the subject has been primarily selected in the PubMed^®^ database (National Institutes of Health) by using keywords such as “ozone” in combination with “nano*” or “liposome”. Investigations on ozonated nanocarriers intended only for biomedical/therapeutic purposes were considered, while articles dealing with ozonated nanoconstructs for environmental, industrial, or waste processing were excluded. No limiting time window was established to select the literature; however, since the application of nanotechnology to O_3_ therapy is a quite recent research field, most of the reviewed articles were published in the last two decades.

## 3. Liposomes

Liposomes are uni- or multi-lamellar spherical vesicles formed by colloidal aggregation of phospholipids in an aqueous medium [69]. Phospholipids are amphiphilic molecules, and, in liposomes, they aggregate as two layers of oppositely oriented molecules, with the polar heads facing outward (at the outer and the inner face of the bilayer), and their hydrophobic tails facing inward. As a consequence, liposomes may be loaded with both hydrophilic molecules, which accumulate in the aqueous core, and lipophilic molecules, which are entrapped inside the lipid bilayer. Thanks to this versatility as well as to their biocompatible, biodegradable, non-toxic, and non-immunogenic nature, liposomes have emerged as efficient delivery systems for many bioactive compounds.

Due to the high stability of O_3_ in unsaturated fatty acids, liposomes have been immediately envisaged as suitable nanocarriers to improve O_3_ delivery and bioavailability in comparison to ozonated oils. Ozonated liposomes may be easily prepared by the well-established thin-film hydration method starting from ozonated vegetable oils, phospholipids, and cholesterol dissolved in a solvent, which is subsequently evaporated [e.g., [70]]. Moreover, since 1995, several liposome-based drug-delivery systems have been approved by the FDA and EMA [71,72,73].

**Figure 1:**
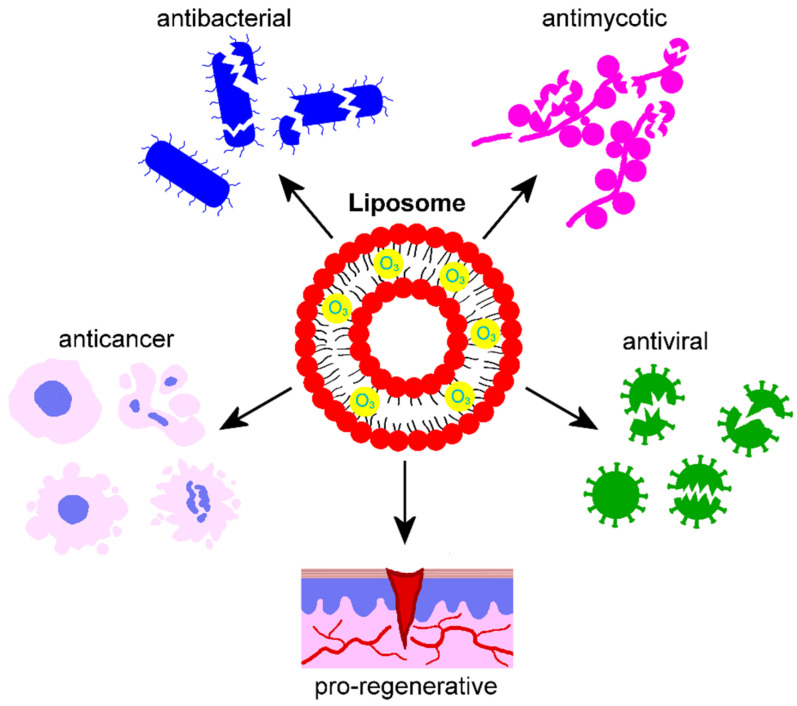
Schematic representation of a liposome loaded with ozonated oil, and the effects documented by the literature [74,75,76,77,78,79,80,81,82,83,84,85,86,87,88,89,90,91].

Based on these advantages, ozonated liposomes have attracted the interest of companies, thus promoting basic and clinical research on their efficacy. Commercially available ozonated liposomal formulations intended for ocular treatment have been investigated for their antiseptic and pro-regenerative efficacy in either in vitro or clinical studies (Figure 1). The antimycotic activity of a formulation based on ozonated sunflower oil in liposomes (Ozodop^®^, FB Vision, San Benedetto del Tronto, Italy) was tested against Candida species of clinical interest (*C. albicans*, *C. glabrata*, *C. krusei*, and *C. orthopsilosis*) in an in vitro study [74]: the significant increase in reactive oxygen species (ROS) induced by the treatment was suggested as the main cause for Candida cell damage and death. The same liposomal formulation proved to be non-toxic in vitro for human cell lines from corneal and conjunctival epithelium, and demonstrated effective antimycotic activity against *C. albicans* and antibacterial activity against various bacterial strains (*Staphylococcus aureus*, *S. epidermidis*, *Moraxella catarrhalis*, *Pseudomonas aeruginosa*, and *Escherichia coli*) [75]. In an in vitro study on bacterial cultures, Ozodop^®^ was found to exert an efficient antibacterial activity not only against twenty strains belonging to *S. pneumoniae*, *S. agalactiae,* and *S. pyogenes* (all known to be widely represented in ocular infections) but also against sixty bacterial strains belonging to *P. aeruginosa*, *S. aureus*, and *S. epidermidis* characterized by antibiotic resistance [76]. Since the formation of microbial biofilm on ophthalmic devices such as contact lens represent a serious problem, Zerillo et al. [77] investigated in vitro the capability of two commercially available formulations of ozonated-sunflower oil in liposomes (Ozodrop^®^ and Ozodrop^®^ gel, FB Vision) to eradicate *P. aeruginosa* and *S. aureus* biofilms from different supports, demonstrating an excellent inhibitory effect on bacterial viability, as well as high efficacy in eradicating the biofilms. Moreover, these liposomal formulations were found to stimulate the expression of antimicrobial peptides, and improve proliferation and migration when applied to a human keratinocyte cell line, suggesting their suitability to treat chronic non-healing infected wounds. Consistently, in an in vitro study, Gentili et al. [78] demonstrated the high efficacy of Ozodrop^®^ in inhibiting bacterial viability of various strains (*S. aureus*, methicillin-resistant *S. aureus*, *S. epidermidis*, *P. aeruginosa*, and *E. coli*), controlling antibiotic resistance and bacterial adhesion, and exerting anti-biofilm activity. These in vitro results were corroborated by a clinical study demonstrating reduced bacterial load in regular contact-lens wearers treated with liposomal sunflower ozonated oil (Ozonest^®^, Esteve, Barcelona, Spain) [79]. Ozodop^®^ gel was also tested in vitro for its effects on a human corneal epithelial cell line and on human explanted corneal tissues treated to mimic eye dryness. In addition, its antiviral efficacy was evaluated in a green monkey kidney-derived cell line (Vero cells) infected with SARS-CoV-2 [80]. The results demonstrated that Ozodop^®^ gel was non-toxic at the lowest concentrations tested, stimulated cell proliferation, restored normal conditions in the eye-dry corneal model, and efficiently inhibited viral replication. Ozodrop^®^ demonstrated antibacterial, anti-inflammatory, and pro-regenerative properties in vivo, when administered to humans, horses, cats, and dogs affected by blepharitis, conjunctivitis, keratitis, or corneal ulcers [81,82]. Similarly, ocular treatment with Ozonest^®^ in patients affected by blepharitis or blepharoconjunctivitis significantly improved the pathological signs and symptoms [83]. Ozodrop^®^ was also suitable for antibacterial prophylaxis of periocular skin and ocular conjunctiva before surgical procedures in dogs [84] and humans [85,86]. The same liposomal formulation reduced signs and duration of viral conjunctivitis, although it was unable to affect the subepithelial corneal infiltrates [87], and to prevent the spread of the viral infection in a SARS-CoV-2-positive patient affected by acute anterior uveitis [88]. Lastly, Ozodrop^®^ was successfully tested in newborns as a safe and antibiotic-sparing alternative for the ocular surface lubrication and antibacterial prophylaxis during screening for prematurity retinopathy [89].

However, research on ozonated liposome-based nanosystems was not only limited to commercially available antiseptic products but was also dedicated to developing anticancer nanotools (Figure 1). Zeng et al. [90] aimed at improving the efficacy of radiotherapy by directly inducing hydroxyl radicals and supplying O_3_, which makes ionizing irradiation more efficient in the hypoxic tumor microenvironment. To do this, they developed a novel O_3_-carrying liposome perfluorodecalin (PFD) nanosystem (they called O_3__PFD@Liposome), which promoted a very high O_3_ solubility (18.4-fold higher than that in water). In detail, O_3_ was concentrated into the PFD, which was then emulsified in nanodroplets; subsequently, by using the thin-film dispersion method, the ozonated PDF droplets were loaded into a phospholipid bilayer membrane made of lecithin and cholesterol, thus obtaining O_3__PFD@Liposome. This nanosystem was tested both in vitro and in vivo. When administered to breast cancer cell lines, O_3__PFD@Liposome was efficiently internalized and, after irradiation, significantly inhibited cell growth by promoting apoptosis and immunogenic cell death. After intravenous injection into mice bearing breast tumor cell xenografts, PFD@Liposome accumulated in the tumor microenvironment entering tumor cells, and the combination of O_3_-fueled irradiation and cell cycle checkpoint blockade synergistically inhibited tumor growth. Recently, Chen et al. [91] developed a nanosystem (O_3_-001@lipo) consisting of liposomes enwrapping O_3_ and RRx-001, a small molecule acting as an inhibitor of CD47 (i.e., the cancer cell surface protein that prevents macrophage phagocytosis). Briefly, a perfluorohexane emulsion (used as O_3_ carrier) was prepared by sonication and added to a mixture of phospholipids (lecithin and cholesterol) and RRx-001 to form 001@lipo by using the thin-film hydration method. Then, O_3_ (50 mg/L) was directly saturated in 001@lipo, thus obtaining O_3_-001@lipo. By administering in vitro O_3_-001@lipo to breast cancer cells, macrophages, and primary myeloid-derived suppressor cells, ROS and reactive nitrogen species were generated, thus inducing extensive immunogenic cell death. O_3_-001@lipo was also intravenously administered to a mouse subcutaneous tumor model and to a mouse mammary orthotopic tumor model: after X-ray irradiation, O_3_-001@lipo showed a potent anti-tumor efficacy by enhancing cytotoxic lymphocyte infiltration and reversing the immunosuppressive action of the tumor microenvironment. This last finding is consistent with the observation in vitro that, after administering O_3_-001@lipo, M2-type tumor-associated macrophages repolarized into the anti-tumor M1-type.

Besides liposomes, some ozonated oil nanoemulsions were experimented with. Nanoemulsions are colloidal systems obtained by mixing oil, an emulsifier, and water; the size of the resulting particles depends on the type and the ratio of the components, as well as on the exerted mechanical and shearing forces. Aydın and Kazancı [92] produced an ozonated olive oil nanoemulsion system by a low-energy method involving titration of distilled water (aqueous phase) into surfactant-oil (organic phase) with constant stirring. This method gave rise to highly stable droplets of about 200 nm in diameter that exerted antibacterial activity against *S. aureus* and *E. coli* in vitro. This nanoemulsion was tested for its anti-tumor potential as a radiosensitizer in vitro, using mouse melanoma cells and human ovarian cancer cells [93]. It was thus demonstrated that, even under low X-ray irradiation, cancer cells treated with the ozonated nanoemulsion underwent cell cycle arrest and ROS-induced DNA damage.

## 4. Nanobubbles

Nanobubble technology is a developing platform aimed at delivering gases through tiny bubbles (less than 200 nm in diameter) formed in an aqueous medium. Produced by various methods (e.g., cavitation, hydrodynamics, electrolysis, nano-pore membranes), nanobubbles are characterized by high specific surface area, high internal pressure, and negative zeta potential, all features providing great stability and longevity (over one month) in water. Moreover, when nanobubbles burst, free radicals are generated (recent reviews in [94,95]), thus promoting per se disinfectant effects. Due to these properties, nanobubbles have attracted attention in biomedical research as promising nanocarriers to enhance stability, biodistribution, and bioavailability of poorly soluble active agents, which are loaded in their core (e.g., [96]). Moreover, the versatility, the capability of gaining access to sites inaccessible to current treatments, and the simple generation in chemical-free environments have made nanobubbles an attractive product for the biomedical market, despite the limited information on their potential adverse effects (e.g., cytotoxicity, release mechanism, and time, clearance/accumulation).

**Figure 2:**
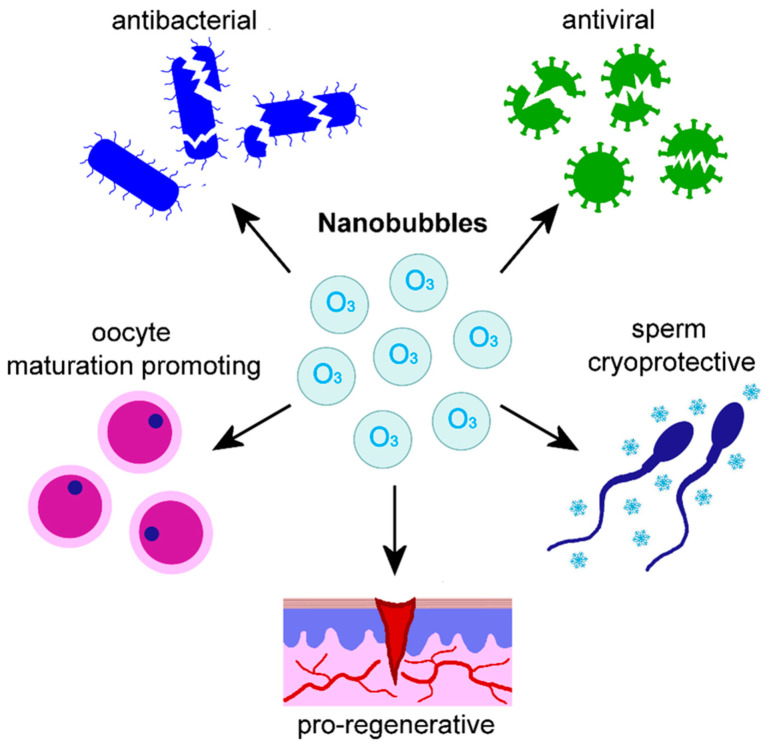
Schematic representation of O_3_ nanobubbles and their effects documented by the literature [97,98,99,100,101,102,103,104,105,106,107,108,109,110,111].

In the specific case of medical O_3_, nanobubbles have been widely investigated for their antiseptic potential (Figure 2). The antibacterial activity of commercially available O_3_ nanobubble water (OPT Creation Inc., Yokohama, Japan) against *E. coli* and *Mycobacterium smegmatis* (one of the most disinfectant-resistant bacteria) was demonstrated after long-term storage (one year at 4 °C [97]). In a clinic study, Hayakumo et al. [98] used a patented procedure to generate O_3_ nanobubble water (JP, 2010-167365, A. 2010-08-05) consisting of collapsing microbubbles in an electrolyte solution by means of a physical stimulus (e.g., electrical discharge, ultra-high temperature and pressure), thus inducing ions concentration around the gas nucleus to form stable nanobubbles of less than 100 nm in diameter. The authors evaluated the effects of irrigation with this O_3_ nanobubble water as an adjuvant periodontal treatment to subgingival mechanical debridement. This O_3_ nanobubble treatment decreased the probing pocket depth (i.e., the distance between the gingival margin and the base of the periodontal pocket), increased the clinical attachment level (the distance between the cemento-enamel junction and the base of the periodontal pocket), and reduced the bacterial population in subgingival plaque. In a subsequent in vitro study, the same research group [99] demonstrated a potent antibacterial activity of O_3_ nanobubble water tested on periodontopathogenic bacteria (*Porphyromonas gingivalis* and *Aggregatibacter actinomycetemcomitans*) and on human buccal and gingival tissue models; notably, no cytotoxicity for human oral tissues was observed. Takizawa et al. [100] set up an O_3_ ultrafine bubble water generator consisting of a micro blender and a cooled polyvinyl chloride tank where distilled water was ozonized by gas insufflation at high pressure. This generator allowed the production of nanobubbles containing high concentrations (4–6 ppm) of O_3_. The authors successfully tested in vitro the inactivating potential of the O_3_ nanobubble water produced with this procedure against various pathogenic bacteria (*Streptococcus pneumoniae*, *P. aeruginosa*, *S. mutans*, *S. sobrinus*, *Fusobacterium nucleatum*, *Prevotella intermedia*, *P. gingivalis*) occurring in the oral cavity and upper airway. O_3_ nanobubble water exerted effective bactericidal activity also against *S. pneumoniae* and *P. aeruginosa* grown on healthcare equipment (toothbrush and gauze), while showing low cytotoxicity toward a human gingival epithelial cell line. Conversely, when commercially available O_3_ nanobubble water (O_3_ NanoGAS^®^ Water, Shinbiosis Corporation, Osaka, Japan) was tested on cultured fibroblasts, a reduction in cell migration and proliferation was found, suggesting that the cytotoxicity of O_3_ nanobubbles may depend on the cell type [101].

Recently, Takahashi et al. [102] produced O_3_ nanobubbles surrounded by a solid shell composed of iron hydroxide. They used a generator where gaseous O_3_ was injected into a plastic tank containing circulating ultrapure water enriched in FeSO_4_ (as bulk nanobubble stabilizer) and MnSO_4_ (to form permanganate ions, thus improving the oxidizing potential of the aqueous solution). The cleaning properties of this O_3_ nanobubble water on titanium dental implants were found to persist for more than one month, suggesting that O_3_ nanobubble water is able not only to remove organic contaminants from the implant surface but also to alter the surface properties, making it hydrophilic, probably due to the adhering nanobubbles.

Besides the antimicrobial activity, O_3_ nanobubble water has been investigated for its regenerative properties (Figure 2). Using a rat model of oral mucositis, Hayashi et al. [103] demonstrated that O_3_ nanobubble water, produced as described in [97], is effective for the treatment of chemotherapy-induced stomatitis: rinsing out the oral cavity with O_3_ nanobubble water decreased bacterial population and improved healing.

A series of clinical cases showed that daily oral rinsing with commercially available O_3_ nanobubble water (NAGA Co., Ltd., Japan) for one to four months was able to cure skin diseases such as palmoplantar pustulosis [104] and pigmented purpuric dermatosis [105,106]. These findings suggested that oral bacteria may be involved in the pathogenesis of these skin diseases since the healing was due to O_3_-induced inactivation of bacteria.

Alkan et al. [107] set up a novel O_3_ formulation referred to as nanobubble liposome solutions. The production procedure, protected by a patent (patent application TR201804452A2), provides that medicinal vegetable oils are supplied with gaseous O_3_ and then converted to liposomes by means of high-speed ultrasonic cavitation and a cold fusion technique of distilled water containing ozonated oil and NaCl. This nanobubble liposome solution had efficient antibacterial activity in vitro against *S. aureus* and *E. coli*, maintaining its effectiveness for over one year. Later on, a nanobubble ozonated hyaluronic acid-decorated liposomal solution, obtained by a procedure similar to [107], with the addition of hyaluronic acid to improve targeting (Sonofarma Pharmaceutical Chemicals Industry and Trade Inc., Umurbey, Nilüfer/Bursa, Turkey; patent applications WO2019240713A2, WO2019240713A3, US2021007360A1, TR201904790A, TR2021-00105), was developed as antiseptic agent. Its antibacterial activity in vitro was verified in cultures of *S. aureus*, *S. pneumoniae,* and *E. coli*, while the anti-viral activity was evaluated on recombinant SARS-CoV-2 copies grown in Vero cells as well as on a SARS-CoV-2 mouse infection model. The formulation proved to be safe both in vitro (on human primary lung epithelial cells, placenta-derived mesenchymal stem cells, and mouse embryonic fibroblast cells) and in vivo (on mice, rabbits, and hamsters) [108]. Subsequently, the nanobubble ozonated hyaluronic acid-decorated liposomal solution (Sonofarma Pharmaceutical Chemicals Industry and Trade Inc.; Umurbey, Nilüfer/Bursa, Turkey; patent application PCT/TR2022/050177) proved to efficiently inhibit in vitro other bacteria (*P. aeruginosa*, *Acinetobacter baumannii*), as well as methicillin-resistant *S. aureus*, and to maintain its effectiveness for at least two years. Moreover, no negative effects were found after both sub-acute and sub-chronic systemic toxicity testing performed in mice and rats [109].

The nanobubble ozonated hyaluronic acid-decorated liposomal solution (Sonofarma Drug and Chemistry Ind. Trade Ltd. Co.; Patent File No PCT/TR2022/050177, WO2022186802A1) was investigated in vitro for the protective effects on ram sperm cryopreservation, demonstrating that adding low doses of this formulation to cryopreservation extenders improved post-thaw and post-incubation sperm motility, and the integrity of plasmalemma, acrosome, and DNA (Figure 2). The O_3_-driven mechanisms accounting for these beneficial effects were identified in the increase in the antioxidant capacity, due to enhanced synthesis of reduced glutathione, and in the rise in mitochondrial membrane potential, which promotes sperm motility [110]. The effect of this O_3_ nanobubble–liposome formulation (Sonofarma Drug and Chemistry Ind. Trade Ltd. Co.; Patent File No PCT/TR2022/050177, WO2022186802A1) was also investigated on in vitro nuclear maturation of canine oocytes [111]: it was demonstrated that, at appropriate doses, the ozonated liposomal formulation improved the oocyte meiotic competence by enhancing the antioxidant defense and stimulating maturation promoting factors (Figure 2).

## 5. Nanoconstructed Hydrogels

Nanoconstructed hydrogels, referred to as nanocomposite hydrogels or nanogels, combine the unique properties of traditional hydrogels with the enhanced functionalities offered by nanomaterials. Traditional hydrogels are three-dimensional networks that can absorb and retain large amounts of water or biological fluids, without dissolving. Their highly hydrated, soft, and porous structure, together with their biocompatibility, biodegradability, and the similarity with the native tissue extracellular matrix, make the nanoconstructed hydrogels excellent candidates for various biomedical applications [112].

Traditional hydrogels often suffer from poor mechanical strength and inadequate functionality, but these limitations may be overcome in nanoconstructed hydrogels by incorporating various nanomaterials into a matrix. These nanomaterials (that typically have at least one dimension in the range of 1 to 100 nanometers) may be combined with a polymeric network, and allow creating hybrid constructs with significantly enhanced and tunable properties that allow tailored and minimally invasive treatments.

**Figure 3:**
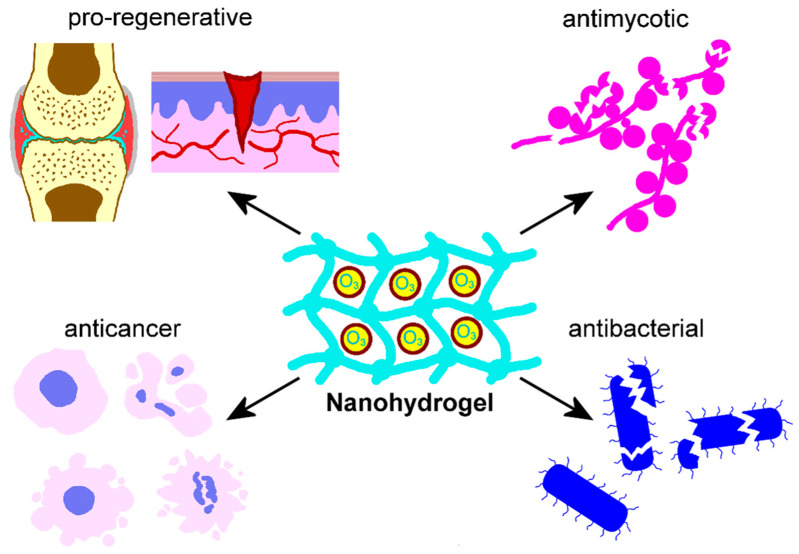
Schematic representation of an ozonated nanoconstructed hydrogel and its effects documented by the literature [113,114,115,116,117].

Extensive research has consistently reported optimal outcomes in the production of nanoconstructed hydrogels for a wide range of biomedical applications, among which dermatology holds a prominent position. Ma et al. [113] designed an O_3_-loaded hydrogel (they named ozonegel) by combining ozonated oil emulsion with a zwitterionic polymer formed by the light-initiated polymerization of the monomer 3-[Dimethyl-[2-(2-methylprop-2-enoyloxy)ethyl]azaniumyl]propane-1-sulfonate. In this network, encapsulated O_3_ enhanced its solubility and extended its lifespan, facilitating the sustained release of both O_3_ and the produced ROS. O_3_ biocompatibility, antibacterial effects, and wound healing efficacy were successfully validated in vitro on bacterial cultures (*E. coli*, *S. aureus*, *P. aeruginosa*, *S. faecium*), and on a mouse fibroblast cell line, as well as in vivo on a murine model of wound healing (Figure 3). In detail, ozonegel significantly reduced the colonies of all the bacteria tested, showed no cytotoxic activity on cultured cells, and significantly accelerated wound closure in mice. Histological analyses confirmed that ozonegel promoted tissue regeneration, stimulating fibroblasts to proliferate and secrete collagen. Furthermore, ozonegel supported a more efficient healing process, exerting a dual modulatory effect on inflammation, boosting it at the early phase of healing and significantly reducing the macrophage activity and the secretion of pro-inflammatory factors (e.g., the tumor necrosis factor-alpha (TNF-α), interleukin (IL)-1β, and IL-6) in later stages.

Lenart-Boroń et al. [114] developed a novel, efficient, cost-effective, and non-toxic bionanocomposite with antimicrobial properties, which may serve as an alternative to antibiotic administration for promoting wound healing. This nanoconstruct consisted of a nano/microencapsulated system incorporating ozonated olive oil within a hyaluronan matrix. Calcium ions were added to the matrix not only as cross-linking agents, but also because of their crucial roles in modulating proliferation, differentiation, and maturation of keratinocytes and fibroblasts, as well as in maintaining the epidermal lipid barrier. This formulation was effective against a variety of Gram-positive and Gram-negative bacteria, including not only common opportunistic and mild pathogens, but also highly pathogenic and antibiotic-resistant strains like Enterococcus and Acinetobacter species. The results obtained make this ozonated nanogel a promising biocomposite to promote tissue regeneration by preventing the wound area from being colonized by pathogenic microorganisms (Figure 3). Similarly, Khachatryan et al. [115] developed an innovative nanoconstructed hydrogel based on hyaluronic acid incorporating micro/nanocapsules of ozonated olive oil. The antimicrobial potential and cytotoxicity of the system was assessed through microbiological analysis and in vitro testing on a human keratinocyte cell line. These nanogels were rheologically stable and displayed a weak-to-moderate antimicrobial effect against both commensal skin bacteria and pathogenic mycetes (Figure 3). Moreover, the nanosystem was not cytotoxic, making it a promising tool in medicine and cosmetology.

O_3_-containing nanoconstructed hydrogels were also proposed as an innovative approach to treat the debilitating degenerative joint disease, osteoarthritis (Figure 3). Wu et al. [116] developed an injectable thermoresponsive hydrogel loaded with an O_3_-rich nanocomposite. In detail, O_3_ was encapsulated into nanoparticles crafted from perfluorotributylamine and fluorinated hyaluronic acid to improve stability; then, this O_3_-rich nanocomposite was loaded into an injectable thermoresponsive hydrogel made of D-mannose and hydroxypropylchitin. In vitro tests on a mouse monocyte/macrophage cell line revealed that the ozonated nanohydrogel significantly reduced the levels of vascular endothelial growth factor (VEGF, a protein highly expressed in osteoarthritis, promoting angiogenesis and cartilage degeneration), pro-inflammatory markers IL-1β, IL-6, TNF-α, and inducible nitric oxide synthase (iNOS). Furthermore, in a mouse chondrogenic cell line, this ozonated nanohydrogel stimulated the expression of collagen II and aggrecan, as well as the proliferation of chondrocytes, suggesting its possible application for cartilage regeneration. Additionally, in vivo studies performed in mice confirmed that the ozonated hydrogels significantly mitigated osteoarthritis by decreasing synovial inflammation, cartilage destruction, and subchondral bone remodeling.

A nanoconstructed hydrogel has also been developed for oncological application (Figure 3). Zhang et al. [117] proposed a thermoresponsive O_3_-enriched spray gel to suppress the tumor recurrence of hepatocellular carcinoma. They demonstrated both in vitro (on a human hepatoma cell line) and in vivo (on mice) that ozonated nanohydrogel induced ferroptosis and apoptosis by altering the expression of relevant genes (e.g., glutathione peroxidase 4, acyl-CoA synthetase long chain family member 4, cyclin-dependent kinase inhibitor 1A) and causing considerable lipid peroxidation. These effects significantly reduced tumor recurrence when the gel was post-surgically applied, thereby prolonging the survival of tumor-bearing mice.

## 6. Other Nanoconstructs for Ozone Delivery

In addition to the nanocarriers detailed above, two types of nanovesicles have been developed for O_3_ delivery: niosomes and polymeric nanoparticles.

Niosomes are amphiphilic nanostructures made of non-ionic surfactants. These are synthetic molecules that, like phospholipids, have a hydrophilic head and a hydrophobic tail that allow them to form a bilayer structure in water [118]. The properties of these vesicles are closely related to the composition, size, lamellarity, volume, surface charge, concentration, and fabrication techniques [119]. Compared to other nanovesicles (such as liposomes), niosomes are generally more stable, cheaper, easier to design, biocompatible, and biodegradable [120]. Moreover, the presence of hydrophilic, amphiphilic, and lipophilic moieties accommodates drug molecules with a wide range of solubility, making these systems suitable for delivering many pharmacological agents [119].

**Figure 4:**
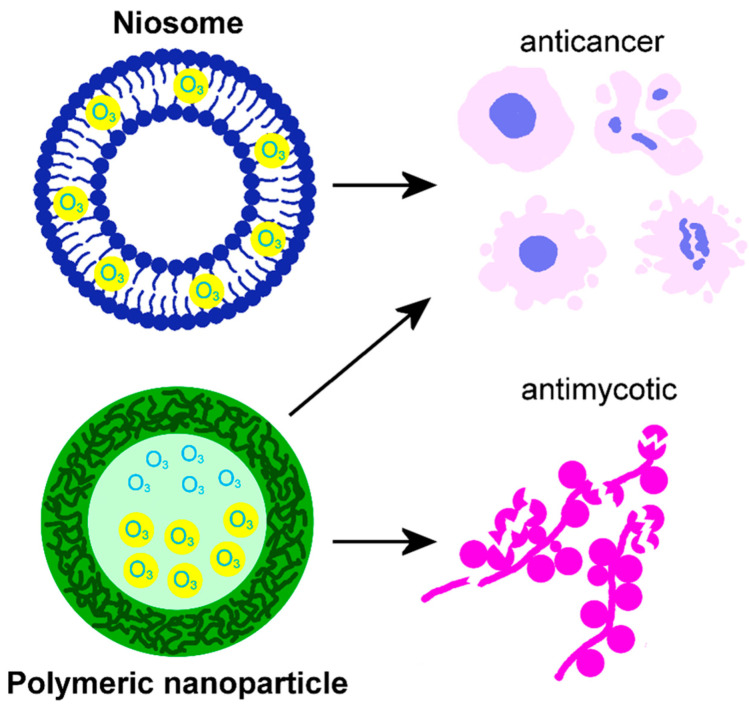
Schematic representation of a niosome and a polymeric nanoparticle loaded with O_3_, and their effects documented by the literature [121,125,126].

Fahmy et al. [121] developed niosomal vesicular nanoplatforms loaded with ozonated olive oil to enhance skin permeation and boost anti-melanoma effects (Figure 4). Niosomes, as nanovesicles, possess a distinctive structure with hydrophilic outer and inner surfaces and a lipophilic intermediate layer, which enables them to encapsulate both water-soluble and hydrophobic components, like olive oil. Encapsulating ozonated olive oil in niosomes significantly improved the O_3_ water solubility, its skin permeation in an ex vivo skin model, and its anticancer activity on human melanoma cells in vitro.

Another type of nanocarrier investigated for O_3_ delivery is polymeric nanoparticles. Based on their internal structure, polymeric nanoparticles are categorized as either nanospheres or nanocapsules [122]. Polymeric nanospheres are made of a solid polymeric matrix where the drug is dispersed. Polymeric nanocapsules have a “core–shell” structure that consists of a polymeric wall that surrounds a liquid/solid core where the active substances are dissolved. This increases the drug-loading efficiency while reducing the polymeric matrix content of the nanoparticle [123]. On the other hand, the polymeric shell provides a physical barrier able to protect the encapsulated drug, control its release, and increase its bioavailability. In addition, the surface of the shell can be easily functionalized with targeting ligands (e.g., antibodies, peptides), thus enabling targeted delivery [124]. Despite the numerous advantages, careful consideration must be given to the choice of polymer, the cost of production, and the stability issues that can reduce their effectiveness and make handling difficult.

Santos et al. [125] developed polymeric nanocapsules made of a thin, polymeric shell covering a core loaded with two active antifungal substances (ozonated oil and terbinafine hydrochloride) for topical application on the skin (Figure 4). On in vitro artificial skin models, these polymeric nanocapsules were able to control the release of O_3_ and the drug, and to improve skin permeation. Moreover, the nanocapsules increased the antimycotic activity of terbinafine hydrochloride against four dermatophyte fungi species (*Trichophyton rubrum*, *T. mentagrophytes*, *Nannizzia gypsea*, and *Microsporum canis*).

Song et al. [126] developed poly(lactic-co-glycolic acid) nanoparticles encapsulating O_3_-enriched perfluorodecalin for application in oncology (Figure 4). The surface of these nanoparticles was properly functionalized to facilitate their targeted delivery to the tumor microenvironment. This innovative carrier was efficiently internalized in triple-negative breast cancer cell lines of murine and human origin, where it allowed for the long-term storage of O_3_. Low-energy microwave irradiation of cells triggered the release of O_3_ from the nanoparticles to the intracellular environment, thus inducing ROS generation and subsequent cytolytic cell death. The tumor-specific neoantigen resulting from dead tumor cells promoted infiltration of cytotoxic T-lymphocytes: this provides a rationale for a therapy based on the immune checkpoint blockade. The administration of these nanoparticles also proved to be an effective supporting therapy in vivo, in a triple-negative breast cancer animal model. In fact, the delivery of O_3_ significantly improved the anti-tumor efficacy of a PD-1 blockade antibody.

## 7. Conclusions

This review demonstrates that the research on nanosystems for delivering medical O_3_ is very active and productive. The main purpose for the development of the various nanosystems described in the literature was O_3_ stabilization and controlled delivery over time, thus overcoming the instability of gaseous O_3_. In addition, nanosystems were designed to make O_3_ administration more effective, e.g., by improving biological barrier permeability or by functionalizing the nanocarrier surface for targeted delivery.

It appears that a limited number of nanoconstruct types have been tested so far for O_3_ delivery. In summary, liposomes were the most investigated nanocarrier type, probably because of the relatively easy production methods, as well as of the possibility to put them on the market as FDA/EMA-approved products (most of the described liposomal systems are commercially available). As for nanobubbles, while the technology for their generation is well-established (nanobubble water used in some of the reviewed articles is commercially available), studies on their application in nanomedicine as delivery systems are still limited. The interest in these innovative nanocarriers may have been limited by the fact that currently, nanobubble-based products have been approved by the FDA and EMA only as ultrasound contrast agents. Other nanosystems such as nanoconstructed hydrogels, niosomes, and polymeric nanoparticles have been scarcely explored to deliver O_3_, although some hydrogels incorporating nanocomponents and polymer-based nanoconstructs have been approved by the FDA and EMA.

Regarding the therapeutic applications of the ozonated nanosystems developed in the reviewed articles, in most cases, they were used as antiseptic agents, although original purposes have also been envisaged. The majority of O_3_-loaded liposomes were investigated for their antibacterial, antimycotic, and antiviral properties when used for topical ocular treatment, also promoting tissue regeneration. Two liposomal systems were designed as radiosensitizers for anticancer treatment, obtaining positive results in vivo. O_3_ nanobubble water was successfully tested as an antibacterial and antimycotic agent for the oral cavity, also showing pro-regenerative properties. More complex O_3_ nanobubble systems, named nanobubble liposome solutions, were developed as antibacterial and antiviral agents for topical and intranasal administration as well as to improve sperm cryopreservation and promote oocyte maturation. Three formulations of nanoconstructed hydrogels designed to deliver O_3_-demonstrated antibacterial properties promoted wound healing. An innovative injectable thermoresponsive hydrogel loaded with O_3_-rich nanocomposites was designed to treat osteoarthritis, giving positive results as an anti-inflammatory and pro-regenerative agent. A thermoresponsive O_3_-enriched spray gel was successfully tested as an anticancer tool. An O_3_-loaded niosomal vesicular nanoplatform was developed to cross the skin barrier and act as an anti-melanoma agent. Two formulations of O_3_-loaded polymeric nanoparticles were designed for topical skin treatment as an antimycotic agent or as an adjuvant anticancer tool, effectively supporting immunotherapy.

It is worth noting that the biomedical research on nanocarriers for O_3_ delivery is a rather new entry in the nanotechnological field, and this explains the limited literature on the subject. In the future, it will be vital to deepen the knowledge of the mechanisms and timing of the O_3_ release by the presently developed nanosystems. At the same time, it may be foreseen that new nanocarrier formulations will be developed to transport O_3_ through tissues and organs. Nanocarriers will specifically be designed to cross the skin, ensuring effective transdermal penetration, or to bypass the blood–brain barrier, reaching the central nervous system, or to resist the harsh conditions of the gastrointestinal tract. Delivering O_3_ to the deep derma or muscle will avoid the direct topical injection of gaseous O_3_, while the rectal administration of O_3_-loaded nanocarriers could allow controlled gas release through the intestinal mucosa to the blood circulation, replacing the more invasive systemic hemotherapy. Gastrointestinal diseases could be treated by oral administration of suitable O_3_-loaded nanoconstructs. Nanosystems suitable for intranasal administration could be used to deliver O_3_ to the nervous tissue, with the aim of treating inflammatory or tumor diseases, avoiding systemic side effects.

The challenge to improve the nanosystem-mediated administration of medical O_3_ has just begun: much work still needs to be undertaken, and new avenues are to be explored.
